# Predição do Mapa de Estresse em Aorta Ascendente: Otimização da Posição Coaxial no Implante Valvar Aórtico Percutâneo

**DOI:** 10.36660/abc.20190385

**Published:** 2020-10-13

**Authors:** Diego Celis, Bruno Alvares de Azevedo Gomes, Ivan Ibanez, Pedro Nieckele Azevedo, Pedro Soares Teixeira, Angela Ourivio Nieckele

**Affiliations:** 1 Pontifícia Universidade Católica do Rio de Janeiro Departamento de Engenharia Mecânica Rio de JaneiroRJ Brasil Pontifícia Universidade Católica do Rio de Janeiro (PUC-Rio) - Departamento de Engenharia Mecânica, Rio de Janeiro, RJ - Brasil; 2 Instituto Nacional de Cardiologia Ministério da Saúde Rio de JaneiroRJ Brasil Instituto Nacional de Cardiologia, Ministério da Saúde, Rio de Janeiro, RJ – Brasil; 3 Fitcente NiteróiRJ Brasil Fitcenter, Niterói, RJ – Brasil

**Keywords:** Estenose da Valva Aórtica/cirurgia, Estenose da Valva Aórtica/diagnóstico por imagem, Comorbidade, Implante de Prótese de Valva Cardíaca/tendências, Ecocardiografia/métodos, Angiotomografia Computadorizada, Resultado do Tratamento

## Abstract

**Fundamento:**

O implante valvar aórtico percutâneo (TAVR, do inglês Transcatheter Aortic Valve Replacement) reduz a mortalidade de pacientes portadores de estenose aórtica grave. O conhecimento da distribuição da pressão e tensão de cisalhamento na parede aórtica pode ajudar na identificação de regiões críticas, onde o processo de remodelamento aórtico pode ocorrer. Neste trabalho é apresentado um estudo de simulação computacional da influência do posicionamento do orifício valvar protético na hemodinâmica na raiz de aorta e segmento ascendente.

**Objetivos:**

A presente análise apresenta um estudo da variação do padrão de fluxo devido a alterações no ângulo do orifício valvar.

**Métodos:**

Um modelo tridimensional foi gerado a partir do exame de angiotomografia computadorizada da aorta de um paciente que foi submetido ao procedimento de TAVR. Diferentes vazões de fluxo foram impostas através do orifício valvar.

**Resultados:**

Pequenas variações no ângulo de inclinação causaram mudanças no padrão de fluxo, com deslocamento na posição dos vórtices, na distribuição de pressão e no local de alta tensão cisalhante na parede aórtica.

**Conclusão:**

Essas características hemodinâmicas podem ser importantes no processo de remodelamento aórtico e distribuição de tensão, além de auxiliar, em um futuro próximo, a otimização do posicionamento da prótese valvar percutânea. (Arq Bras Cardiol. 2020; [online].ahead print, PP.0-0)

## Introdução

Durante muitos anos, a substituição da valva aórtica por cirurgia de “peito aberto” era o tratamento padrão para casos de estenose aórtica grave,^1 - 3^ reduzindo sintomas e aumentando a sobrevida.^4 - 7^ No entanto, alguns pacientes de alto risco não devem ser submetidos à cirurgia convecional,^8 , 9^ seja pela idade avançada, disfunção ventricular esquerda, ou presença de múltiplas comorbidades.^10 - 12^ Para essa classe de pacientes, uma alternativa, menos invasiva, foi desenvolvida em 2002,^13^ chamada de implante de valva aórtica transcateter (TAVR, do inglês *Transcatheter Aortic Valve Replacement* ).^14^

Quando realizado pelo procedimento cirúrgico convencional, o implante valvar é preciso, porém invasivo. No procedimento TAVR, a prótese é liberada na região do anel aórtico, substituindo a valva disfuncionante sem removê-la, por meio de cateteres e auxílio de imagens fluoroscópicas. Contudo, o método está sujeito a maior variabilidade no posicionamento da prótese, dada a natureza do procedimento.^15 , 16^ Além disso, a presença de calcificações excêntricas no ânulo aórtico pode impedir a total expansão da prótese percutânea, afetando, assim, o posicionamento coaxial da prótese após sua liberação.^17^

O posicionamento da valva pode ser definida com base na relação entre o orifício e o ânulo, com sua inclinação definida como o ângulo entre a linha central do ânulo aórtico e a linha central do orifício protético. Variações na composição bem como no posicionamento da prótese (posição coaxial da prótese aórtica) em relação à valva nativa do paciente pode gerar mudanças hemodinâmicas significativas na raiz aórtica, tais como na intensidade de turbulência, direção do fluxo e maior queda de pressão. Sabe-se que variações no fluxo sanguíneo na aorta ascendente estão relacionadas ao processo de remodelamento aórtico e condições patológicas, tais como dilatações, aneurismas e tortuosidades.^18 , 19^ A identificação de altos níveis de tensão cisalhante e pressão é importante dada a sua associação com a dilatação aneurismática da aorta ascendente.^20^

O padrão helicoidal do fluxo sanguíneo, antes e após o paciente ter sido submetido ao procedimento de TAVR, varia consideravelmente pelo efeito da geometria da prótese implantada, sua inclinação e posição final.^21^ Atualmente, pouco se sabe acerca das consequências hemodinâmicas da ausência de coaxialidade da prótese percutânea. Essas variações não são totalmente conhecidas, e é de grande interesse analisar a influência deste procedimento sobre o remodelamento aórtico, para melhorar seu projeto e processo de acoplamento. Assim, no presente trabalho, realizou-se um estudo para investigar a influência de pequenas variações no ângulo coaxial valvar sobre o fluxo sanguíneo no interior da aorta.

A definição do padrão de fluxo aórtico utilizando o exame de angiotomografia computadorizada (ATC) em vez de procedimentos invasivos pode ajudar a definir a melhor estratégia terapêutica. Este poderá ser considerado uma boa prática na assistência em saúde e mais um passo em direção à medicina de precisão.

## Métodos

Para melhor representar a geometria da aorta, um modelo vascular foi construído a partir de uma ATC da aorta sincronizada com eletrocardiograma (ECG-gated) realizada antes da TAVR em um paciente do sexo masculino de 77 anos de idade. O paciente apresentava disfunção sistólica leve do ventrículo esquerdo e estenose aórtica degenerativa grave com classe funcional III ( *New York Heart Association* , NYHA). A prótese implantada foi uma Edwards-SAPIEN. O paciente forneceu consentimento livre e esclarecido prévio para participar do estudo, o qual foi registrado no Conselho Nacional de Ética em Pesquisa (Ministério da Saúde, Brasil) e aprovado pelo Comitê de Ética em Pesquisa do Instituto Nacional de Cardiologia.

A ATC foi realizada em um tomógrafo de 64 canais (Somatom Sensation 64, Siemens, Alemanha). Uma série de cortes foi selecionada, que incluiu desde o ânulo aórtico até a aorta torácica. As imagens DICOM foram transferidas para o software FIJI para a segmentação da região desejada da aorta e estudo da fase sistólica do ciclo cardíaco. A segmentação de uma ATC antes do implante é uma extrapolação válida, uma vez que não existe diferença significativa entre dados de ATC antes e após a cirurgia. O diâmetro D efetivo da prótese valvar aórtica foi determinado a partir de medidas do ecocardiograma transtorácico no pós-operatório, utilizando a equação da continuidade de massa.

Apesar do ciclo cardíaco ser naturalmente transiente, o foco do presente trabalho é o período sistólico, quando as paredes da aorta estão distendidas, fornecendo o diâmetro máximo, com pequena variação dada a complacência vascular. Além disso, a prótese valvar aórtica se abre completamente em um intervalo de tempo muito curto, alcançando o diâmetro efetivo D muito rapidamente. Assim, para analisar a influência do posicionamento da prótese aórtica sobre o campo de fluxo e distribuição das tensões, foram feitas algumas simplificações do modelo.

1. A superfície da aorta foi considerada rígida, *i.e* ., sua complacência foi desconsiderada. Tal aproximação é menos conservadora, uma vez que, devido à complacência, a pressão dentro da aorta encontra-se reduzida em caso de dilatação.
2. A valva foi posicionada na região da entrada, no centro do ânulo aórtico. Os folhetos da prótese valvar aórtica não foram modelados. No pico sistólico, os folhetos encontram-se completamente abertos, resultando em um orifício com diâmetro efetivo *D* . As artérias coronárias também não foram incluídas no modelo devido ao baixo fluxo através delas no pico sistólico. Essas simplificações foram introduzidas devido a custo-efetividade da simulação do modelo, e acreditamos que estas não possuem um impacto significativo sobre os resultados de velocidade de fluxo no pico sistólico.
3. O escoamento foi modelado em regime permanente, correspondendo ao momento do pico sistólico, o que pode ser considerado como condição crítica (vazão máxima).^22^ Essa aproximação permite determinar a tensão média e distribuição de velocidade. No entanto, o índice de cisalhamento oscilatório, o qual está associado à degeneração aneurismática,^23^ não pode ser determinado.
4. Os efeitos de gravidade foram desconsiderados uma vez que as variações de pressão são dominantes.
5. Segundo Sun e Chaicana,^24^ o sangue pode ser considerado fluido newtoniano, *i.e* ., sua tensão de cisalhamento é diretamente proporcional à taxa de deformação do elemento de fluido. Essa aproximação pode ser aplicada se a taxa de cisalhamento for maior que 100s^- 1^ .^25 , 26^ Ainda, sob condições normais a 36^o^C, o sangue pode ser considerado um fluido incompressível, com viscosidade constante.^27 , 28^
6. No pico sistólico (vazão máxima), o jato que flui pelo orifício valvar é turbulento. Segundo estudos anteriores, de escoamentos hemodinâmicos turbulentos, a turbulência foi determinada com o modelo de média de Reynolds. Com base em uma comparação entre dados numéricos e experimentais,^33^ foi escolhido o modelo de turbulência κ–ω SST,^34^ recomendado para situações de baixo número Reynolds.

Com base nas hipóteses apresentadas acima, o campo de escoamento através da aorta pode ser obtido pela solução das equações médias de Reynolds de Navier-Stokes com:

$$\left(1\right)\frac{\partial u_{i}}{\partial x_{i}}\;=\;0\;;\;\frac{\partial {\mathrm{\rho u}}_{j}u_{i}}{\partial x_{j}}\;=\;-\frac{\partial \hat{p}}{\partial x_{i}}\;+\;\frac{\partial }{\partial x_{j}}\;\left[\left(\mu \;+\mu_{t}\;\right)\;2S_{\mathrm{ij}}\right]\;;\;S_{\mathrm{ij}}\;=\;\frac{1}{2}\;\left(\frac{\partial u_{i}}{\partial x_{j}}\;+\frac{\partial u_{j}}{\partial u_{i}}\right)$$

onde x_i_ representa os eixos das coordenadas e u_i_ o componente de velocidade média no tempo; ρ é a densidade, $\hat{p}\;=p\;+\;2/3\;\rho \kappa$ é a pressão modificada, que inclui a pressão dinâmica turbulenta, a qual inclui a pressão dinâmica turbulenta (κ é a energia cinética turbulenta);µ e µ_t_ são a viscosidade molecular e a viscosidade turbulenta; µ_t_ é determinada com base na solução das equações diferenciais para a energia cinética turbulenta κ e a taxa específica de dissipação ω.^34^

**Figura 1:**
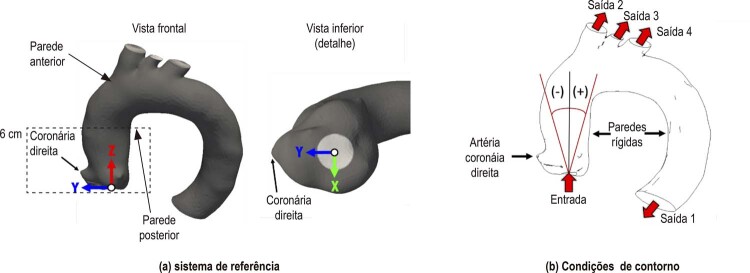
– *Domínio computacional: sistema de referência e condições de contorno.*

A Figura 1 ilustra esquematicamente o domínio computacional correspondente à aorta. O contorno externo do domínio computacional é a camada mais interna (íntima) da aorta, que será aqui chamada apenas de “parede” da aorta. O sangue entra na aorta através da prótese, com orifício efetivo de 1,54 cm^2^ de área, na base da raiz aórtica ( Figura 1a ). O plano da entrada coincide com o plano x-y, e perpendicular à coordenada axial z. O ângulo de inclinação θ da valva é definido em relação ao eixo z, onde um θ negativo está em direção à coronária direita, e positivo para a parede posterolateral da aorta ( Figura 1b ).

A vazão volumétrica Q é definida na entrada. Segundo Ku D.N.,^35^ para esta situação em que o número Womersley é alto (>10), pode-se considerar um perfil uniforme para os componentes de velocidade, bem como para as quantidades de turbulência. Com base nos dados de Gomes B.A.A.,^36^ 10% de intensidade de turbulência na entrada foi recomendado.

O fluxo deixa a aorta por quatro saídas, como ilustrado na Figura 1b. Em todas as regiões da saída, aplicou-se uma condição de fluxo difusivo nulo e a vazão foi dividida entre elas, conforme recomendado por Alastruey et al.,^37^ e Nardi et al.,^38^ com base nos valores médios encontrados no corpo humano. Saída 1 (aorta descendente): 69,1%; saída 2 (tronco braquiocefálico): 19,3%; saída 3 (artéria carótida comum esquerda): 5,2% e saída 4 (artéria subclávia esquerda): 6,4%.

Na superfície da aorta, uma condição de não escorregamento foi definida como condição de contorno. A condição de contorno κ na superfície sólida também é zero, e a dissipação específica nas paredes (ω_W_) é definida com base na espessura da subcamada molecular.^34^

Uma vez que o escoamento foi modelado como incompressível, o nível de pressão é irrelevante e, assim, a distribuição da pressão foi determinada em relação à pressão na valva aórtica, p_in_.

### Modelagem numérica

As equações de conservação de massa, quantidade de movimento linear e de turbulência, que caracterizam o problema foram resolvidas com o programa ANSYS Fluent v17,0, baseado no método de volumes finitos.^39^ Uma malha com 400 mil nós foi definida para todos os casos. A malha foi delineada com base teste de independência da malha, realizado para se garantir a qualidade da solução na região da entrada da valva e na parede aórtica, com distância adimensional da parede ao primeiro nó, $y^{+}\;=\;{\mathrm{\rho u}}_{\tau }y/\mu$ , menor que 4,5 na superfície da aorta, conforme recomendado para o modelo κ – ω SST. Aqui, $u_{\tau \;}=\;\sqrt{\tau_{w}}/\;\rho$ é a velocidade de fricção, onde $\tau_{w}\;=\;\mu \partial u\;/\;\partial n\;{|}_{w}$ é a tensão de cisalhamento da parede (baseado no gradiente normal na parede). A malha definida forneceu variação da queda de pressão menor que 0,3% na região ascendente da aorta, indicada na Fig. 1a , quando a malha foi duplicada.

## Resultados

A influência do ângulo de inclinação sobre a velocidade axial, pressão e tensão de cisalhamento na parede aórtica foi aqui avaliada. Com base em um estudo prévio,^36^ seis diferentes ângulos da valva de entrada foram analisados: -4°, -2°, 0°, 1°, 3° e 5°. Considerou-se a situação mais crítica correspondente ao pico sistólico, i.e., vazão máxima durante o período de sístole (25 L/min).

Para visualização dos campos internos, foi selecionado um plano central com 6 cm de altura e orientado em relação à artéria coronária direita ( Figura 1a ). De acordo com a posição do plano central escolhido, a parede esquerda do plano corresponde à parede anterior da aorta e a parede direita corresponde à parede posterior.

Para a análise da distribuição do cisalhamento nas paredes, examinou-se a geometria completa, apesar de termos dado ênfase à parede contra a qual o jato sitólico incide (parede anterolateral direita da aorta ascendente).

**Figura 2:**
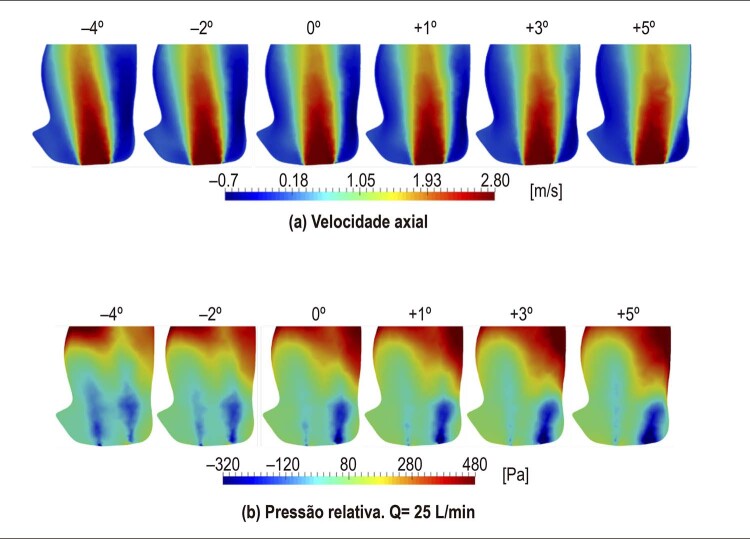
– *Velocidade axial e pressão relativa nos diferentes ângulos de inclinação.*

A Figura 2 compara, para todos os ângulos de entrada estudados, os isocontornos do componente de velocidade axial (u_z_) e pressão relativa (p – p_in_) no plano central da aorta ( Figura 1 ). Pode-se observar um deslocamento progressivo do campo de velocidade axial com a variação do ângulo valvar de entrada, sem que haja uma mudança substancial do diâmetro do jato. Quando o jato é desviado para a esquerda (ângulos negativos), este atinge a parede anterior da aorta. Além disso, uma região com velocidade negativa para a direita do jato é identificável, indicando a presença de recirculação. Por outro lado, a inclinação valvar para a direita (ângulos positivos) desloca o jato para longe da parede anterior, aproximando-se da parede posterior da aorta. O jato se espalha, e uma região menor de velocidades negativas ocorre no lado posterior da aorta. À medida que o jato de entrada atinge a superfície da aorta, a pressão aumenta substancialmente, induzindo um fluxo descendente. Observa-se uma mudança na localização das áreas de alta pressão, as quais estão localizadas na parede anterior em ângulos de inclinação negativos e se movem para a parede posterior em ângulos de inclinação positivos.

**Figura 3:**
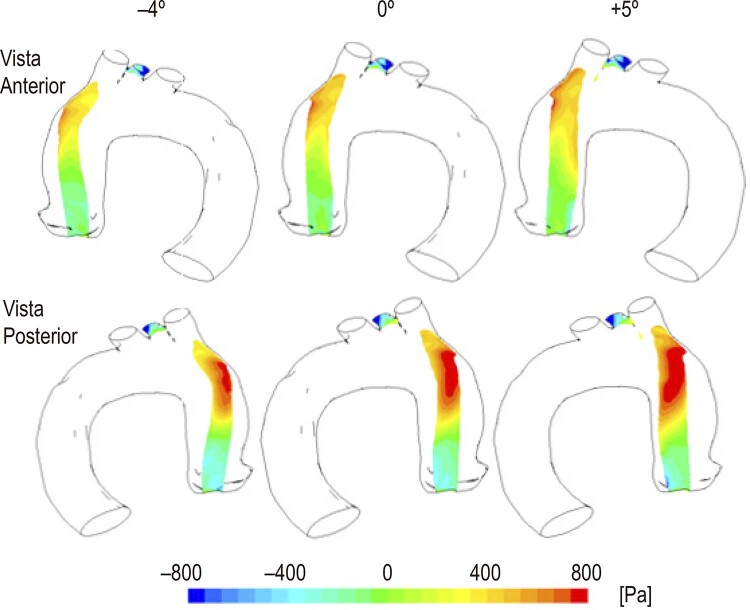
– *Influência do ângulo de inclinação da válvula. Isosuperfície de u_*z*_ =1,3 m/s, colorida segundo pressão relativa Q=25L/min.*

Para três ângulos representativos (-4°, 0° e +5°), a Figura 3 apresenta uma isosuperfície correspondente a um componente de velocidade axial constante, u_z_ =1,3 m/s. A superfície é colorida pela pressão relativa. Para visualizarmos melhor o fluxo, são apresentadas imagens da vista anterior e posterior. Para os três ângulos de inclinação, o jato de entrada atinge o lado esquerdo da parede aórtica, onde a pressão atinge seu valor máximo. Pela curvatura da parede da aorta, o jato se curva em direção ao arco aórtico. Para o ângulo negativo (direção oposta à curvatura da aorta), pode-se observar uma curvatura mais acentuada do jato. Para o ângulo de inclinação positivo, o jato de entrada encontra-se mais alinhado com o formato da aorta e o jato é mais vertical.

**Figura 4:**
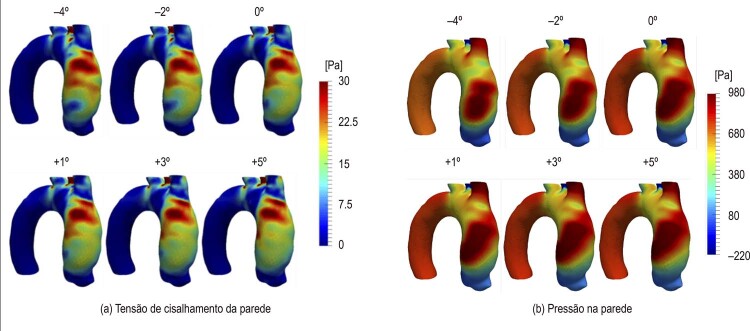
– *Influência do ângulo de inclinação da válvula sobre a tensão de cisalhamento e pressão da parede da aorta. Q=25L/min.*

Na Figura 4 , a tensão de cisalhamento da parede e a pressão na parede da aorta são apresentadas para seis ângulos e Q=25 L/min. A aorta é visualizada de modo a focar a região onde os maiores efeitos ocorrem e, neste caso, ocorre na parede anterolateral direita da aorta ascendente. Pode-se ver claramente que a região de alto cisalhamento corresponde à parede anterolateral direita da aorta ascendente. Foram obtidos valores de até 30 Pa, como relatado por vários outros autores.^40 - 42^ Esses valores de elevadas tensões de cisalhamento na parede estão concentrados em uma região próxima ao tronco braquiocefálico. Analisando a figura, pode-se perceber que, quando o ângulo muda de valores negativos para valores positivos, ocorre um deslocamento e redução dos valores mais altos de tensão de cisalhamento, mostrando que a região de alta pressão corresponde à região onde o jato de entrada atinge a parede da aorta. Também pode-se observar que as pressões mais altas ocorrem na região anterior para os casos de ângulos negativos. À medida em que os ângulos aumentam e se tornam positivos, a região de maior pressão é deslocada para a zona posterior. Isso implica um deslocamento e diminuição do estresse mecânico na parede da aorta ascendente pela mudança da inclinação da valva protética em direção à parede posterior.

**Figura 5:**
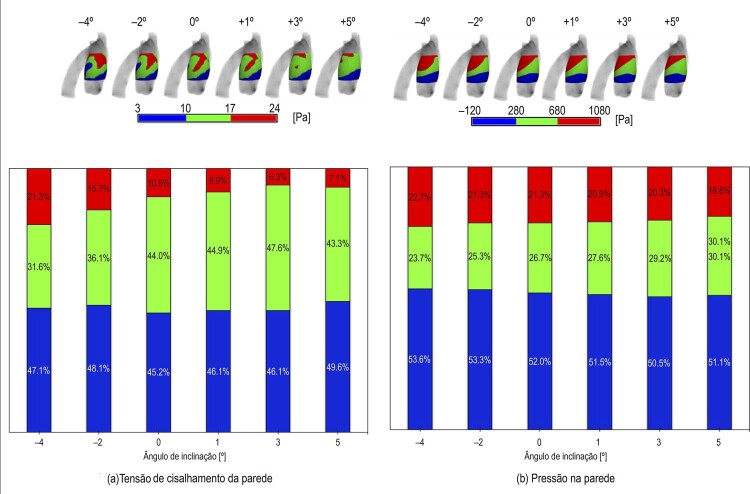
– *Identificação da área de alta tensão de cisalhamento e pressão, com distribuição da porcentagem na parede anterolateral, em função do ângulo de inclinação; Q=25L/min.*

Para melhor identificação da região da aorta ascendente com elevada tensão de cisalhamento e pressão, definiu-se uma sub-região crítica (correspondente à parede anterolateral direita, Fig. 1a ) onde os principais efeitos ocorrem. Essa região foi considerada como referência para a análise. Adicionalmente, foram definidos três intervalos de tensão de cisalhamento e pressão, onde a cor azul corresponde a valores mais baixos, a cor verde a valores intermediários e o vermelho a valores mais altos. Analisando a Figura 5 , pode-se notar uma redução significativa no tamanho da região com alta tensão de cisalhamento quando a inclinação do fluxo aumenta de -4^o^ a +5^o^. Apesar de se observar uma redução da área de alta pressão com o aumento do ângulo de posição valvar, essa redução é bem menos marcante. Para determinar a variação do tamanho da região com valores altos de tensão (cisalhamento e pressão), a porcentagem da área superficial coberta por cada intervalo de tensão em relação à área de referência foi determinada ( Figura 5 ). Observa-se que o tamanho da zona de baixa tensão de cisalhamento tende a permanecer constante em um valor de aproximadamente 47%, enquanto o tamanho da zona de alta tensão de cisalhamento diminui progressivamente com a variação do ângulo de inclinação. A mudança de pressão pela inclinação do ângulo valvar é relativamente pequena, com muita pouca variação no tamanho da região com valores de alta pressão.

**Figura 6:**
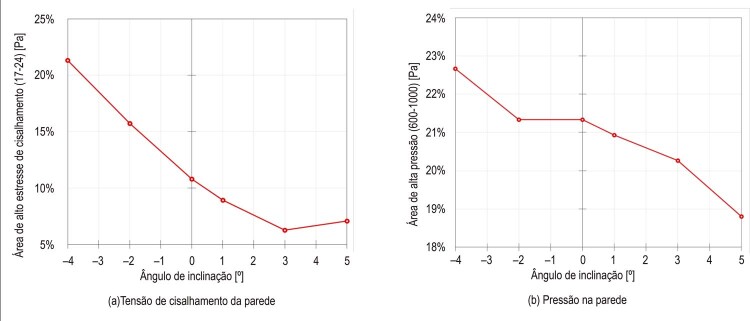
– *Porcentagem da área (parede anterolateral direita da aorta ascendente) com valores elevados de tensão de cisalhamento e pressão com a variação no ângulo de inclinação.*

Na Figura 6 , pode-se observar uma redução de até 15% no tamanho da área com valores mais altos de tensão de cisalhamento, quando o ângulo de fluxo muda de -4° a +3°. A influência do ângulo de fluxo sobre o tamanho da área de alta pressão é muito menor, com uma redução de apenas 6%, com o aumento do ângulo de entrada.

## Discussão

Os resultados deste estudo mostram que o ângulo de inclinação da prótese valvar induz mudanças nos padrões hemodinâmicos da aorta. No entanto, em todos os casos, o jato tende a atingir a parede lateral esquerda da aorta ascendente. Ângulos negativos de inclinação provocam um desvio do jato em direção à parede anterior, sem uma modificação substancial do diâmetro do jato, considerando os valores da posição central. Essa mudança concentra a pressão e a tensão de cisalhamento nessa parede, aumentando seu estresse mecânico.

Quando a prótese assume uma angulação positiva, o jato inclina-se para a parede posterior, com um ligeiro aumento no diâmetro. Essa variação no ângulo alivia o estresse mecânico na parede anterior da aorta ascendente, diminuindo e deslocando os valores mais altos de tensão de cisalhamento em todas as paredes da aorta.

Apesar da presente análise limitar-se à anatomia de apenas um paciente, ela fornece uma perspectiva de uma grande variação no comportamento do fluxo devido apenas a modificações no ângulo, sem influência de outro viés, como o formato da aorta.

O impacto significativo da inclinação da prótese valvar sobre as propriedades hemodinâmicas do fluxo aórtico faz com que recomendemos aos fabricantes que considerem esse parâmetro no projeto das próteses percutâneas. Ainda, em um futuro próximo, podemos sugerir que um estudo da influência dos ângulos de inclinação da prótese, na hemodinâmica do fluxo aórtico, seja realizado com cada paciente antes de ser submetido ao procedimento de TARV. Sabe-se que cada paciente apresenta diferenças na geometria aórtica e na resistência da parede da aorta e, por isso, tal análise deve ser individualizada. O estudo poderá contribuir para a implementação da TAVR, ao recomendar ajustes estratégicos no posicionamento das próteses valvares e assim prevenir elevado estresse mecânico, o qual pode ter influências no processo de remodelamento aórtico.
