# Supplementary material for: Predição do Mapa de Estresse em Aorta Ascendente: Otimização da Posição Coaxial no Implante Valvar Aórtico Percutâneo
Source: Arq Bras Cardiol. 2020 Oct 13;115(4):680–7. [Article in Portuguese] doi: 10.36660/abc.20190385 (PMC8386968; doi:10.36660/abc.20190385)
Supplement: Translation

# Translation: Prediction of Stress Map in Ascending Aorta - Optimization of the Coaxial Position in Transcatheter Aortic Valve Replacement

**Authors:** Diego Celis, Bruno Alvares de Azevedo Gomes, Ivan Ibanez, Pedro Nieckele Azevedo, Pedro Soares Teixeira, Angela Ourivio Nieckele

**Affiliations:** 1 Brazil Pontifícia Universidade Católica do Rio de Janeiro (PUC-Rio) - Departamento de Engenharia Mecânica, Rio de Janeiro, RJ – Brazil; 2 Brazil Instituto Nacional de Cardiologia, Ministério da Saúde, Rio de Janeiro, RJ – Brazil; 3 Brazil Fitcenter, Niterói, RJ – Brazil

**Keywords:** Aortic Valve Stenosis/surgery, Aortic Valve Stenosis/diagnostic imaging, Comorbidity, Heart Valve Prosthesis Implantation/trends, Echocardiography/methods, Computed Tomography Angiography/methods Treatment Outcome

## Abstract

**Backgroud:**

Transcatheter aortic valve replacement (TAVR) can reduce mortality among patients with aortic stenosis. Knowledge of pressure distribution and shear stress at the aortic wall may help identify critical regions, where aortic remodeling process may occur. Here a numerical simulation study of the influence of positioning of the prosthetic valve orifice on the flow field is presented.

**Objective:**

The present analysis provides a perspective of great variance on flow behavior due only to angle changes.

**Methods:**

A 3D model was generated from computed tomography angiography of a patient who had undergone a TAVR. Different mass flow rates were imposed at the inlet valve.

**Results:**

Small variations of the tilt angle could modify the nature of the flow, displacing the position of the vortices, and altering the prerssure distribution and the location of high wall shear stress.

**Conclusion:**

These hemodynamic features may be relevant in the aortic remodeling process and distribution of the stress mapping and could help, in the near future, the optimization of the percutaneous prosthesis implantation. (Arq Bras Cardiol. 2020; [online].ahead print, PP.0-0)

## Introduction

For many years, the open-chest aortic valve replacement was the standard-of-care treatment for cases of severe aortic stenosis,^1 - 3^ reducing symptoms and improving survival.^4^ However, some high-risk patients cannot undertake open-chest surgery,^8 , 9^ either because of their advanced age, left ventricular dysfunction, or the presence of multiple coexisting conditions.^10 - 12^ For this class of patients, in 2002^13^ a powerful less invasive alternative was developed, called transcatheter aortic valve replacement (TAVR).^14^

When performed by open surgical procedure, valve placement is precise, but invasive. In the TAVR procedure, prosthesis is released in the region of the aortic annulus, replacing the damaged valve without removing it, through the use of catheters and with the aid of fluoroscopic images. However, the method is subject to greater variability in the positioning of the prosthesis, due to the nature of the procedure.^15 , 16^ Also, the presence of eccentric calcifications in the aortic annulus may avoid the complete expansion of the percutaneous prosthesis, thereby affecting its coaxial position after the release process.^17^

The valve position can be defined based on the relationship between its effective orifice and the annulus, with its inclination determined as the angle formed between aortic annulus centerline and the effective orifice centerline. Variations in prosthesis composition as well as in its positioning (coaxial position of the aortic prosthesis) in relation to the patient’s native valve can generate significant hemodynamic changes in the aortic root, such as the turbulence intensity, flow direction and pressure drop increase. It is well known that blood flow variations in the ascending aorta are related to the aortic remodeling process and pathological conditions, such as dilatation, aneurysmal formations and tortuosity.^18 , 19^ Identification of high shear stress and pressure is important due to their association with aneurysmal dilatation of the ascending aorta.^20^

The helical patterns of blood flow, before and after a patient have undergone a TAVR procedure, vary considerably by the effect of the geometry of the prosthesis implanted, its inclination and final positioning.^21^ Currently, little is known about the hemodynamic consequences of the lack of coaxiality of the percutaneous prosthesis. These variations have not been completely understood and it is of great interest to analyze the influence of this procedure on aortic remodeling, to improve its design and assembly. Therefore, in the present work, a study is performed to investigate the influence of small variations in the coaxial angle of the valve on the flow field inside the aorta.

The definition of the aortic flow pattern based on computed tomography angiography (CTA), without using an invasive procedure, may help to define the best care strategy. This could be considered as a good practice in health care and maybe a step further on direction of precision medicine.

## Methods

To better represent the aorta geometry, a vascular model was constructed from a pre-TAVR electrocardiogram gated-scan CTA of the aorta from a 77-year-old male patient. The patient had mild systolic left ventricular dysfunction, and severe degenerative aortic stenosis with New York Heart Association functional class III. The valve implanted was an Edwards-SAPIEN. The patient provided a free, prior and informed consent for participation in the study, which was registered in the National Council of Ethics in Research (Ministry of Health - Brazil) and approved by the Research Ethics Committee, National Institute of Cardiology.

The CTA was performed on a 64-slice scanner (Somatom Sensation 64, Siemens, Germany). A series of CTA slices were selected, covering from the aortic annulus to the thoracic aorta. The DICOM images were transferred to the FIJI software, in order to allow the segmentation of the desired aortic region and study of the systolic phase of the cardiac cycle. Segmentation of a pre-implant CTA is a valid extrapolation, since there is no major difference between pre- and post-operative CTA data. The effective diameter *D* of the aortic prosthesis was determined from post-operative transthoracic echocardiogram measurements, using the continuity equation.

Although the cardiac cycle is naturally transient, the focus of the present work is the systolic period, when the aortic walls are distended, providing their maximum diameter, with small variation due the vascular complacency. Further, the aortic prosthesis completely opens in a very short time interval, reaching its effective diameter *D* very fast. Thus, to analyze the influence of the positioning of the aortic valve on the flow field and stress distribution, a few simplifications of the model were made:

1. The aortic surface was considered rigid, *i.e* ., its complacency was neglected. This approximation is less conservative, since due to complacency, the pressure inside the aorta is reduced in aortic dilatation.
2. The valve was placed at the inlet region, centered in the aortic annulus. The leaflets of the aortic prosthesis were not modeled. At systolic peak, they are completely open, resulting in an orifice with the effective diameter *D* . The coronary arteries were also not included in the model because of the low flow through them at systolic peak. These simplifications were introduced due to the cost-effectiveness of model simulation, and we believe that they do not have a significant impact on the results of peak systolic flow rate.
3. The flow was modeled in steady state, corresponding to the moment of systolic peak, which can be considered as the critical condition (maximum flow rate).^22^ This approximation allows inferring the time average stress and velocity distribution. However, the oscillatory shear index, which is associated to aneurysmal degeneration,^23^ cannot be determined.
4. Gravity effects were neglected since the pressure variations are dominant.
5. According to Sun and Chaichana,^24^ blood can be considered as a Newtonian fluid, i.e., the viscous stress is directly proportional to the fluid element deformation rate. This approximation can be applied if the shear rate is above 100 s^- 1^ .^25 , 26^ In addition, under normal conditions at 36°C, the blood can be considered as an incompressible fluid, with constant viscosity.^27 , 28^
6. At the systolic peak (maximum flow rate), the jet flow leaving the valve orifice is turbulent. Following previous studies on turbulent hemodynamic flows,^29^ the turbulence was determined with the Reynolds-Average model. Based on a comparison between numerical and experimental data,^33^ the turbulence model κ–ω SST,^34^ which is recommended for low Reynolds number situations, was selected.

Based on the hypothesis presented above, the flow field through the aorta can be obtained by the solution of the Reynolds-Averaged Navier-Stokes equations:

$$\left(1\right)\frac{\partial u_{i}}{\partial x_{i}}\;=\;0\;;\;\frac{\partial {\mathrm{\rho u}}_{j}u_{i}}{\partial x_{j}}\;=\;-\frac{\partial \hat{p}}{\partial x_{i}}\;+\;\frac{\partial }{\partial x_{j}}\;\left[\left(\mu \;+\mu_{t}\;\right)\;2S_{\mathrm{ij}}\right]\;;\;S_{\mathrm{ij}}\;=\;\frac{1}{2}\;\left(\frac{\partial u_{i}}{\partial x_{j}}\;+\frac{\partial u_{j}}{\partial u_{i}}\right)$$

where x_i_represents the coordinate axes and u_i_the time-average velocity component; ρ is the density, $\hat{p}\;=p\;+\;2/3\;\rho \kappa$ is the modified pressure, which includes the turbulent dynamic pressure (κ is the turbulent kinetic energy); µ and µ_t_are the molecular and turbulent viscosity; µ_t_is determined based on the solution of the differential equations for the turbulent kinetic energy κ and the specific rate of dissipation ω.^34^

Figure 1 illustrates schematically the computational domain corresponding to the aorta. The outer boundary of the computational domain is the inner layer (intima) of the aorta, which will be referred here simply as aortic wall. The blood enters the aorta through the prosthesis, with an effective orifice area of 1.54 cm^2^, at the base of the aortic root ( Figure 1a ). The inlet plane is coincident with the plane x-y, and perpendicular to the axial z coordinate. The tilt angle θ of the valve is defined in relation to the z-axis, where negative θ is in the direction of the right coronary artery, and positive to the posterolateral aortic wall ( Figure 1b ).

**Figure 1:** – *Computational domain: system of reference and boundary conditions.*

The volumetric flow rate Q is defined at the entrance. According to Ku D.N.,^35^ for the situation under consideration, since the Womersley number is high (>10), a uniform profile for the velocity components, as well as for the turbulent quantities is reasonable. Based on the data of Gomes B.A.A.,^36^ 10% of turbulent intensity was prescribed at the inlet.

The flow leaves the aorta through four exits, as illustrated in Figure 1b , with null diffusive flux. The flow rate was split at the outflow regions, based on average values found in the human body, following the recommendation of Alastruey et al.^37^ , and Nardiet al.^38^ , Output 1 (descending aorta): 69.1%; Output 2 (brachiocephalic trunk): 19.3%; Output 3 (left common carotid artery): 5.2% and Output 4 (left subclavian artery): 6.4%.

At the aortic surface, a non-slip condition was defined as a boundary condition. The boundary condition of κ at the solid surface is also zero, and the specific dissipation in the walls (ω_W_) is defined based on the thickness of the molecular sublayer.^34^

Since the flow was modeled as incompressible, the pressure level is irrelevant, thus, the pressure distribution was determined in relation to the pressure at the aortic valve, p_in_.

### Numerical modeling

The conservation of mass, momentum, and turbulence equations that characterize the problem were solved with ANSYS Fluent software v17.0, based on the finite volume method.^39^ A mesh with 400,000 nodes was defined for all cases. The mesh was designed based on a mesh independence test, performed to guarantee the quality of the solution in the valve inlet region and at the aorta wall, with the dimensionless wall distance of the first node, $y^{+}\;=\;{\mathrm{\rho u}}_{\tau }y/\mu$ smaller than 4.5 at the aortic surface, as recommended for the κ – ω SST model. Here, $u_{\tau \;}=\;\sqrt{\tau_{w}}/\;\rho$ is the friction velocity, where $\tau_{w}\;=\;\mu \partial u\;/\;\partial n\;{|}_{w}$ is the wall shear stress (WSS) (based on the normal gradient at the wall). The defined mesh provided variation of the pressure drop at the ascending aorta region, indicated in Fig. 1a , inferior to 0.3%, when the mesh was doubled.

## Results

The influence of the tilt angle on the axial velocity, pressure and WSS was evaluated here. Based on a previous study,^36^ six different inlet valve angles were analyzed: -4°, -2°, 0°, 1°, 3° and 5°. The most critical situation corresponding to the systole peak, *i.e* ., maximum flow rate during the systole period (25 L/min) was considered.

To visualize the internal fields, a central plane with 6cm of height and oriented with respect to the right coronary artery ( Fig. 1a ) was selected. According to the position of the chosen center plane, the left wall of the plane corresponds to the anterior wall of the aorta and the right wall corresponds to the posterior wall.

To analyze the stress distribution on the walls, the complete geometry was examined, although emphases were given to the wall where the inlet jet impinges (right anterolateral wall of the ascending aorta).

Figure 2 compares, for all inlet angles studied, the isocontours of the axial velocity component (u_z_) and relative pressure (p – p_in_) at the central plane of the aorta ( Figure 1 ). It can be seen a progressive displacement of the axial velocity field with the variation of the inlet valve angle, without substantial modification of the jet diameter. When the jet is tilted to the left (negative angles), it reaches the anterior aortic wall. Furthermore, a region with negative velocity to the right of the jet is identifiable, indicating the presence of a recirculation. On the other hand, the inclination of the valve to the right (positive angles) displaces the jet away from the anterior wall, approaching the posterior aortic wall. The jet undergoes a spreading, and a smaller region of negative velocities occurs at the posterior side of the aorta. As the inlet jet impinges the aorta surface, the pressure increases substantially, and a downward flow is induced. Note a change in the location of the high-pressure areas, which are located at the anterior wall at negative tilt angles and move to the posterior wall at positive tilt angles.

**Figure 2:** – *Axial velocity and relative pressure at different tilt angles.*

For three representative angles (-4°, 0° and +5°), Figure 3 presents an iso-surface corresponding to the constant axial velocity component, u_z_=1.3 m/s. The surface is colored by the relative pressure. To better visualize the flow, front and back views are presented. For the three tilt angles, the inlet jet impinges at the left side of aortic wall, where the pressure reaches its maximum value. Due to the aortic wall curvature, the jet is bent in direction of the aortic arch. For the negative angle (opposite direction than the aortic curvature), a stronger curvature of the jet can be observed. For the positive tilt angle, the inlet jet is more aligned with the aortic shape, and the jet is more vertical.

**Figure 3:** – *Influence of tilt angle of the valve. Iso-surface of u_*z*_ =1,3 m/s, colored by relative pressure. Q=25L/min.*

In Figure 4 , the WSS and the pressure at the aortic wall are shown for six angles and Q=25L/min. The aorta is visualized in such a way as to focus on the region where the greatest effects occur, which in this case occurs in the right anterolateral wall of the ascending aorta. It can be clearly seen that the high stress region corresponds to the right anterolateral wall of the ascending aorta. WWS values up to 30 Pa were obtained, as also observed by several authors.^40 - 42^ This high WSS values are concentrated in a region near the brachiocephalic trunk. Analyzing the figure, it can be perceived that when the angle is modified from negative values to positive values there is a displacement and a reduction of the higher values of WSS, showing that the region of high pressure corresponds to the region where the inlet jet impinges the aortic wall. It can also be seen that higher pressures occur in the anterior region for the negative angles cases. As the angles increase and become positive, the higher-pressure region is displaced to the posterior zone. This implies a displacement and decrease of mechanical stress on the ascending aortic wall by modifying the inclination of the prosthetic valve on the direction of the posterior wall.

**Figure 4:** – *Influence of the tilt angle of the valve on shear stress and pressure at aortic wall. Q=25L/min.*

To better identify the region of the ascending aorta surface with elevated WSS and pressure, a critical sub-region (corresponding to the right anterolateral wall, Fig. 1a ) where the major effects occur was defined. This region was taken as reference to the analysis. Further, three subranges of WSS and relative pressure values were defined, where blue corresponds to lower values, green to intermediate values and red to higher values. Analyzing Figure 5 , it can be seen a significant reduction in the size of the region with high WSS when the flow inclination increases from -4^o^to +5^o^. Although a reduction of the area with high pressure is also observed when the valve position angle is increased, the reduction is much less striking. To determine the variation of the size of the region with high stress values (WSS and pressure), the percentage of superficial area covered by each stress range in relation to the reference area was determined ( Figure 5 ). Note that the size of the low WSS zone tends to remain at a constant value of approximately 47%, while the size of the high WSS zone is progressively reduced by varying the tilt angle. Pressure variation due to valve inclination is relatively small, with very small changes in the size of the region with high-pressure values.

**Figure 5:** – *Identification of the area with high wall shear stress and pressure, with percentage distribution on the anterolateral wall, as a function of the tilt angle, Q=25L/min.*

In Figure 6 , it is possible to observe reduction up to 15% of the size of area with the highest values of WSS, when the flow angle changes from -4° to +3°. The influence of the flow angle on the size of the area with high pressure is much smaller, with a reduction of only 6% with the increase of the inlet angle.

**Figure 6:** – *Percentage of the area (right anterolateral wall of the ascending aorta) with high wall shear stress and high-pressure values by changes in the tilt angle.*

## Discussion

From the results of this study, it was observed that the tilt angle of the prosthetic valve induces changes in the hemodynamic patterns of the aorta. However, in all cases, the jet tends to hit the right lateral wall of the ascending aorta. Negative tilt angles incline the jet towards the anterior wall, without a substantial modification of the jet diameter considering the values of the central position. This change concentrates the pressure and WSS on this wall, increasing its mechanical stress.

As the prosthetic valve takes positive angulation, the jet tilts toward the posterior wall, with a small widening of the jet diameter. This angle variation relieves the mechanical stress on the anterior wall of the ascending aorta, decreasing and displacing higher WSS values in all aortic walls.

Although the present analysis is limited to only one patient, it provides a perspective of great variation in the flow behavior due angle changes, without influence of other bias like the aortic shape.

The significant impact of the inclination of the prosthetic valve on the hemodynamic properties of the aorta flow leads us to recommend that manufacturers consider this parameter in the design of percutaneous prosthesis. One can also suggest, in the near future, that a hemodynamic study of the influence of the tilt angles of the prosthesis should be performed on each candidate before being submitted to the TARV procedure. It is known that each patient has differences in the aortic geometry and in the aortic wall resistance; therefore, such analysis should be individualized. The study could contribute to the implementation of TAVR, by recommending strategic adjustments in the positioning of prosthetic valves, thereby preventing high mechanical stress, which can influence the aortic remodeling process.
